# A Semi-Linear Elliptic Model for a Circular Membrane MEMS Device Considering the Effect of the Fringing Field

**DOI:** 10.3390/s21155237

**Published:** 2021-08-02

**Authors:** Mario Versaci, Alessandra Jannelli, Francesco Carlo Morabito, Giovanni Angiulli

**Affiliations:** 1 DICEAM Department, “Mediterranea” University, I-89122 Reggio Calabria, Italy; morabito@unirc.it; 2 MIFT Department, University of Messina, I-98166 Messina, Italy; ajannelli@unime.it; 3 DIIES Department, “Mediterranea” University, I-89122 Reggio Calabria, Italy; giovanni.angiulli@unirc.it

**Keywords:** membrane MEMS devices, fringing field, semi-linear elliptic problems, numerical methods for BVPs

## Abstract

In this study, an accurate analytic semi-linear elliptic differential model for a circular membrane MEMS device, which considers the effect of the fringing field on the membrane curvature recovering, is presented. A novel algebraic condition, related to the membrane electromechanical properties, able to govern the uniqueness of the solution, is also demonstrated. Numerical results for the membrane profile, obtained by using the Shooting techniques, the Keller–Box scheme, and the III/IV Stage Lobatto IIIa formulas, have been carried out, and their performances have been compared. The convergence conditions, and the possible presence of ghost solutions, have been evaluated and discussed. Finally, a practical criterion for choosing the membrane material as a function of the MEMS specific application is presented.

## 1. Introduction

The development of embedded technologies in the last decade has been mainly due to microdevices, which can manage the connection between the physical layer of a particular problem and the logic of the machine language [1,2,3,4,5,6]. In this context, the interest in both static and dynamic MEMS devices is high [7,8,9]. In particular, a lot of effort have been made concerning dynamic investigations into the response of resonant microbeam [10] as much as micro-resonators [11,12]. This because they provide an excellent approximation of the human–machine interface in all the cases wherein miniaturized-integrated electromechanical systems are required [13,14,15,16,17,18,19,20,21]. Since the first batch device was produced in 1964 [22], the scientific and technological development in the field has strongly influenced the analysis and synthesis of physical-mathematical models that can describe the extremely complex underneath MEMS multi-physics [23,24,25,26,27,28,29]. However, such theoretical models hardly provide analytical solutions. Hence, it appears necessary to have conditions that can ensure both the existence and uniqueness of the solution [23,24,25,26,30] to avoid ghost solutions when the model is solved numerically [30,31,32]. Since the aforementioned analytical solutions are unobtainable, suitable numerical procedures have to be selected, and it becomes imperative to evidence the pros and cons of each method [25,26,30,31,32]. MEMS is a rampant technology employed for realizing thermo-elastic systems [1,33,34,35,36,37], and it has a wide variety of applications ranging from biomedical engineering to microfluidics [38,39,40,41,42]. Moreover, many researchers are actively engaged in the development of important experimental research works for the development and prototyping of special MEMS such as, for example, circular graphene membrane MEMS devices [43,44], SiN circular membrane MEMS devices [45,46], and CMOS MEMS-based membrane-bridge devices [47] particularly useful for industrial applications. Moreover, the scientific community is intensively working on the analysis/synthesis of multi-physical models characterized by a high degree of symmetry, because these are more easily achievable from a technological point of view [24,30,48,49,50,51]. Accordingly, we have focused our attention on a 2*D* circular membrane MEMS device, a kind of geometry widely used in many industrial applications [24,25,48,52]. The membrane deformation is described by its profile, u(r), r∈[0,R] with *r* being the radial coordinate and *R* the radius of the device. As it is well known, for a circular geometry, the physical-mathematical model describing the membrane curvature assumes the following semilinear elliptic form [1,53]:

(1) $$\left\{\begin{array}{c} \Delta u\left(r\right)=-\frac{\lambda^{2}}{{\left(1-u\left(r\right)\right)}^{2}}\\ u\left(r\right)=0,\;\;\;u^{\prime }\left(0\right)=0,\;\;\;0<u\left(r\right)<d \end{array}\right.$$

where $\lambda^{2}$ is a parameter related to the external electrical voltage *V* and *d* is the distance between the parallel disks. (1) is a theoretical model disregarding the fringing field phenomenon [54,55,56,57,58] that occurs when d≪R [17]. A more precise analytic model, from which (1) is derived, is the following [59]:

(2) $$\left\{\begin{array}{c} \Delta u\left(r\right)=-\frac{\lambda^{2}}{{\left(1-u\left(r\right)\right)}^{2}}+\lambda^{2}F\left(u\left(r\right),u^{\prime }\left(r\right),\delta ,\ldots \right)\\ u(-L)=u(L)=0. \end{array}\right.$$

where *F* is a suitable function describing the effects close to the MEMS edges, and δ≥0 is a term that weighs the effects of the fringing field. It is apparent that (1) derives directly from (2) omitting the additional term $F(u\left(r\right),u^{\prime }\left(r\right),\delta ,\ldots )$ under the hypothesis that the effect due to its presence is negligible. However, this hypothesis turned out to be false despite using uniform approximation for the electrostatic field E. On the basis of this premise, we employed the so-called corner-corrected theory, developed in [59], for our analysis, thus exploiting the “corner-corrected model”; more precisely, following the work in [59], the term $F(u\left(r\right),u^{\prime }\left(r\right),\delta ,\ldots )$ in (2) can be written as

(3) $$\lambda^{2}\delta {\left|\nabla u\left(r\right)\right|}^{2}.$$

Now, considering that Δu(r) in (1) has only the radial component, (1) specializes as [48]

(4) $$\left\{\begin{array}{c} u^{\prime \prime }\left(r\right)=-\frac{1}{r}u^{\prime }\left(r\right)-\frac{\lambda^{2}\left(1+\delta \right|u^{\prime }{\left(r\right)|}^{2}}{{\left(1-u\left(r\right)\right)}^{2}}\\ u\left(R\right)=0,\;\;\;\;u^{\prime }\left(0\right)=0,\;\;\;0<u\left(r\right)<d \end{array}\right.$$

(It can be noticed that if δ=0, no fringing field occurs and (4) reduces to (1)). At this stage, we point out that the term $\delta |u^{\prime }{\left(r\right)|}^{2}$ becomes appreciable only if the term $|u^{\prime }\left(r\right)|$ assumes relevant values, a real possibility at the edge of the device in most cases (see Theorem 1 in [23]). Once the membrane deforms, the electrostatic capacitance of the device, $C_{el}$, varies since the distance between the membrane and the upper disk is not constant. Moreover, as physically

(5) $$\frac{\lambda^{2}}{{\left(1-u\left(r\right)\right)}^{2}}\propto {\left|E\right|}^{2}$$

it follows that

(6) $$\frac{\lambda^{2}}{{\left(1-u\left(r\right)\right)}^{2}}=\theta {\left|E\right|}^{2},\;\;\;\;\;\theta \in R^{+}.$$

As proved in [48], the field E is locally orthogonal to the straight-line tangent to the membrane profile itself, so that it is natural to consider |E|∝K(r,u(r)) with K(r,u(r)) mean curvature of the membrane [24]:

(7) $$K\left(r,u\left(r\right)\right)=\frac{1}{2}\left(u^{\prime \prime }\left(r\right)+\frac{1}{r}u^{\prime }\left(r\right)\right).$$

Considering both (6) and (7), (4) can be rewritten as

(8) $$\left\{\begin{array}{c} u^{\prime \prime }\left(r\right)=-\frac{1}{r}u^{\prime }\left(r\right)-\frac{4{\left(1-u\left(r\right)-d^{*}\right)}^{2}}{\theta \lambda^{2}\left(1+\delta \right|u^{\prime }\left(r\right){|}^{2})}\\ u\left(R\right)=0,\;\;\;\;u^{\prime }\left(0\right)=0,\;\;\;0<u\left(r\right)<d, \end{array}\right.$$

which is a 2*D* second-order differential semi-linear elliptic model without explicit nonlinearity. The term $d^{*}$, the so-called critical security distance, guarantees that the membrane does not touch the upper disk. Note that the factor θ, by (6), is an important parameter because it takes into account the electrical properties of the membrane. This last differential model is exceedingly difficult to solve analytically. Accordingly, we focus our attention on obtaining conditions that ensure both the existence and uniqueness of its solutions. Before proceeding with our analysis, we point out how our study is framed in a broader line of research. The first paper was that in [24] in which a 2*D* nonlinear second-order differential model for electrostatic circular membrane MEMS devices (wherein the singularity was not explicitly evident) was studied, starting from (1), from which the following chain of proportionality,

(9) $$\lambda^{2}/{\left(1-u\left(r\right)\right)}^{2}\propto {\left|E\right|}^{2}\propto K^{2}\left(r,u\left(r\right)\right)$$

was proved. As the model studied in [24] did not allow to obtain an explicit analytical solution, the existence was derived through the use of two auxiliary functions satisfying particular properties. In this way, an algebraic condition on the product $\theta \lambda^{2}$, depending on both $d^{*}$ and *V*, was provided. However, as far as uniqueness is concerned, it has not been guaranteed [24]. In [60], stable numerical approaches for recovering the membrane profile, based on the three-stage Lobatto formulas, have been exploited, thus obtaining the ranges for operative parameters and the areas of applicability of the device avoiding the ghost solutions. In contrast, in [25], the equilibrium configurations were analyzed. Finally, in [48], the model was improved and rewritten. In particular, in this study, the effect of the fringing field on membrane curvature has been considered. Furthermore, the study into the stability of the equilibrium configurations, the analysis of the range of possible values for *V*, and the study on the device’s optimal control was conducted.

The main results presented in this paper can be summarized as follows:

1. A new algebraic condition governing the uniqueness of the solution for (8), depending on the electromechanical properties of the membrane material, has been demonstrated. Unlike the 1*D* geometry, this new condition cannot guarantee both the existence and uniqueness of the solution for the model (8).
2. Shooting procedure, Keller-box scheme, and III/IV Stage Lobatto IIIa formulas have been employed, and their numerical performances, related to the membrane profile recovering task, when δ varies in the range of its possible values, have been compared. Furthermore, the values of the parameter $\theta \lambda^{2}$ ensuring the procedures’ convergence have been determined.
3. Ghost solutions have been investigated for obtaining the values of the factor $\theta \lambda^{2}$ that ensures the convergence of each considered numerical procedure, avoiding the ghost solutions’ computation.
4. Finally, the relationship among the numerical convergence criteria, the parameter $\theta \lambda^{2}$, and the intended use of the device has been highlighted.

The paper is organized as follows. In Section 2, the 2*D* electrostatic circular-membrane MEMS device considered in our analysis has been mentioned. Section 3 discusses the curvature-dependent |E| for modeling the 2*D* circular membrane MEMS device with the fringing field. In Section 4 and Section 5, we recall well-known results of the existence and uniqueness for the problem under study. Section 6 suggests a new result concerning the uniqueness of the solution, which depends on the electromechanical properties of the material constituting the membrane, whose proof is reported in the Appendix A. In Section 7, we derive a new condition ensuring both the existence and uniqueness of the solution. In Section 8, we present and discuss the numerical results conducted in our study for recovering the MEMS membrane profile. Finally, in the last section, our conclusions are given.

## 2. A Description of the 2*D* Electrostatic Circular-Membrane MEMS Device

### 2.1. The Point of View of the Actuator

**Figure 1:**
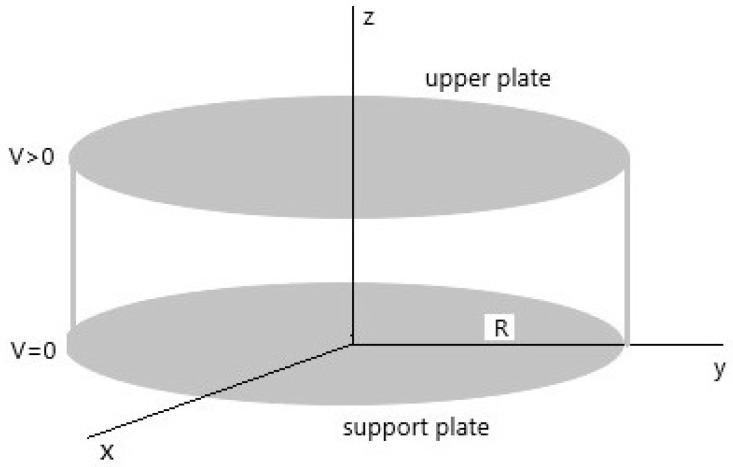
A 2*D* electrostatic circular membrane MEMS device whose metal plates (upper and support ones) are displayed in gray. Between them, a circular membrane, clumped to the edges of the support plate, deforms towards the upper plate without touching it to avoid unwanted electric discharges.

In the usual 3*D* Euclidean space, $R^{3}$, we consider a system of orthonormal Cartesian axes Oxyz, where the *z* axis represents the vertical axis (see Figure 1). The device consists of two parallel circular metal disks with radius *R*, located at a distance of *d* from each other. The lower disk is located on the xy plane, and its center coincides with the origin of the system Oxyz. The device is subjected to an external electrical voltage *V*, and the lower disk is fixed at the potential V=0. A circular membrane with radius *R* is anchored to the edges of the lower disk. It deforms toward the upper disk when a voltage *V* is applied. If we denote with *r* (0<r≤R), the radial coordinate, we have that the membrane profile, u(r), results to be a function of *r*. To overcome its mechanical inertia, the applied voltage *V* must assume values such that the corresponding value of the electrostatic field E inside the device can generate an appropriate electrostatic pressure equal to $p_{el}=0.5\epsilon_{0}{\left|E\right|}^{2}$ (with $\epsilon_{0}$, the permittivity of free space). The term $p_{el}$ can be translated into an equivalent electrostatic force, $f_{el}$, computable as [1,25,48,61]

(10) $$f_{el}=0.5\frac{\epsilon_{0}\pi R^{2}V^{2}}{{\left(d-u\left(r\right)\right)}^{2}},$$

which deflects the membrane, thus achieving a displacement in its center, $u_{0}$, equal to $R^{2}p_{el}/4T$ (where *T* is the radial mechanical tension of the membrane when it is at rest) [1,48]. Electrostatically, if the membrane deforms, the field E, which depends on the distance between the membrane and the upper disk, results to be locally orthogonal to the tangent line to the membrane at the same point [2]. Moreover, the electrostatic capacitance, $C_{el}$, is variable as the distance between the membrane and the upper disk varies locally. We can also observe that the greater the |E|, the greater the curvature of the membrane. On this basis, we have that |E| can be considered locally proportional to the mean curvature K(r,u(r)) of the membrane [23,24,25,30].

The device geometry being such that L≪d, we have that the effect due to the fringing field cannot be neglected [48]. Accordingly [1],

(11) $$\lambda^{2}=\frac{\epsilon_{0}V^{2}{\left(2R\right)}^{2}}{2d^{3}T}=\rho V^{2}$$

in which $\epsilon_{0}$ is the permittivity of the free space.

**Remark** **1.** *$\lambda^{2}$, being T-dependent, is a parameter that expresses the mechanical properties of the membrane. Therefore, $\theta \lambda^{2}$ expresses the electromechanical properties of the membrane.*

### 2.2. The Point of View of the Sensor

In the following, it will be convenient to exploit some similarities with the circular plate MEMS transducer model subject to mechanical pressure *p*.

As is known, the study of the deformation of membranes in MEMS devices starts from considering metal plates subject to mechanical pressure *p* whose deflections *u* satisfy [1,62]:

(12) $$\rho hu_{tt}-T\Delta u+D\Delta^{2}u=0$$

where ρ is the density of the material constituting the deformable plate, and *h* and *T* are the thickness and mechanical tension, respectively. Moreover, indicating the Young’s modulus with *Y* and the Poisson ratio with ν, the stiffness coefficient *D* assumes the following form [62]:

(13) $$D=\frac{Yh^{3}}{12(1-\nu^{2})}$$

Furthermore, if the plates are circular, *u* only depends on *r* so that in the steady-state condition results [1,62],

(14) $$u\left(r\right)=\frac{R^{4}}{64D}{\left(1-{\left(\frac{r}{R}\right)}^{2}\right)}^{2}p$$

However, if r=0, it follows that $u_{0}=R^{4}p/64D$, and from (14),

(15) $$u\left(r\right)=u_{0}{\left(1-{\left(\frac{r}{R}\right)}^{2}\right)}^{2}p$$

In this case, the device works as a transducer. In fact, *p* deforms the membrane and generates u(r)≠0 profile (except at the device’s boundaries). Then, $C_{el}$ becomes [1,62]

(16) $$C_{el}\left(u_{0}\right)=\int_{0}^{R}\frac{2\epsilon_{0}\pi r}{d\left(1-\frac{u(r)}{d}\right)}dr,\;\;\;\mathrm{with}\;\;\left|u_{0}\right|\ll d.$$

As both *h* and *D* are bounded values, u(r) becomes unobtrusive. Therefore, the distance between the two plates remains almost constant and equal to *d*. Moreover, exploiting the Taylor series and the value of $C_{el}$ at equilibrium, $C_{0}=\epsilon_{0}\frac{\pi r^{2}}{d}$, for p=0, (16) can be written as

(17) $$C_{el}\left(u_{0}\right)\approx C_{0}\left(1+\frac{u_{0}}{3d}+\frac{u_{0}^{2}}{5d^{2}}\right)$$

by which it is possible to obtain the electrostatic charge of the membrane, the co-energy of the system, and the electrostatic force. Finally, it is also possible to obtain

(18) $$\left|E\left(r\right)\right|\approx \frac{V}{d-u_{0}{\left(1-{\left(\frac{r}{R}\right)}^{2}\right)}^{2}}.$$

**Remark** **2.** *The physical quantities involved here clearly depend on d, because the circular plate had a significantly high value of D; further, u(r) appeared extremely limited so that any dependency on d−u(r) could be replaced by the dependence on d (which is mathematically more straightforward and, therefore, easier to manage).*

If we consider a membrane instead of the deformable plate, *h* can be neglected. *D* then decreases substantially (see (13)) in the case in which a deformable plate is considered. Bearing in mind that the smaller the *D* value, the more flexible the membrane is, it follows that $u_{0}$ becomes higher with an increased risk of the membrane accidentally touching the upper disk. If the membrane replaces the deformable disk, u(r) takes the form [1]

(19) $$u\left(r\right)=u_{0}\left(1-{\left(\frac{r}{R}\right)}^{2}\right)$$

and

(20) $$u_{0}=\frac{pR^{2}}{4T}$$

with $f_{el}$ as formulated in (10).

**Remark** **3.** *To calculate $f_{el}$ and $p_{el}$ for evaluating the surface of the membrane, $\pi R^{2}$ was considered as if the membrane was at rest. This approximation can be justified because d≪R such that the surface of the deformed membrane is approximately equal to the surface of the membrane at rest.*

The parameters *p* and $p_{el}$ are linked to each other because applying *V* produces the field E in the device which, in turn, generates $p_{el}$, deforming the membrane toward the upper plates. Moreover, indicating by $k_{1}=\frac{R^{2}}{4T}$, from (20), we can write

(21) $$u_{0}=k_{1}p.$$

We can assume that, in our case, *p* comes exclusively from $p_{el}$ due to |E| inside the device. Thus, *p* depends on $p_{el}$ so that the following chain of equalities have to be considered valid:

(22) $$u_{0}=k_{1}p=k_{1}k_{2}p_{el}=kp_{el}.$$

As we will see below, *k* plays an important role in formulating the algebraic condition that governs the existence of the solution for (8) (see, inequality (45)).

**Remark** **4.** *When the membrane is at rest, the distance between the membrane and the upper disk is d. Therefore, $C_{el}$ along any vertical plane $\bar{\sigma }$ whose support is the straight line*

(23) $$\bar{r}:\left\{\begin{array}{c} x=0\\ y=0, \end{array}\right.$$

*with fringing field phenomenon, is [54]*

(24) $${\left(C_{el}\right)}_{C}=\frac{2\epsilon_{0}R}{d}\left\{1+\frac{d}{2\pi R}\ln\left(\frac{2\pi R}{d}\right)\right\},$$

*where C represents the curve that arises from the intersection $\bar{\sigma }$ and the membrane. Therefore, the total $C_{el}$, ${\left(C_{el}\right)}_{total}$ can be written as follows:*

(25) $${\left(C_{el}\right)}_{total}=\int_{0}^{\pi }B\left(\phi \right){\left(C_{el}\right)}_{C}d\phi ={\left(C_{el}\right)}_{C}\int_{0}^{\pi }B\left(\phi \right)d\phi .$$

*where B(ϕ) is a bounded and continuous electrostatic function based on ϕ [63]. Therefore,*

(26) $$\int_{0}^{\pi }B\left(\phi \right)d\phi =\bar{D}<+\infty ,$$

*so that* (25) *becomes*

(27) $${\left(C_{el}\right)}_{total}={\left(C_{el}\right)}_{C}\bar{D}=\frac{2\epsilon_{0}R\bar{D}}{d}\left\{1+\frac{d}{2\pi R}\ln\left(\frac{2\pi R}{d}\right)\right\}.$$

## 3. The Mathematical Model

The model (1), with fringing field phenomenon, becomes

(28) $$\left\{\begin{array}{c} \Delta u\left(r\right)=-\frac{\lambda^{2}{\left(1+\delta |\nabla u\left(r\right)\right|}^{2})}{{\left(1-u\left(r\right)\right)}^{2}}\\ u\left(R\right)=0,\;\;\;\;u^{\prime }\left(0\right)=0,\;\;\;0<u\left(r\right)<d. \end{array}\right.$$

Moreover, the radial symmetry in it with respect to vertical axes r=0 allows to write

(29) $$\Delta u\left(r\right)=\frac{1}{r}u^{\prime }\left(r\right)+u^{\prime \prime }\left(r\right),$$

and considering $\nabla u\left(r\right)=u^{\prime }\left(r\right)$, we can write (28) as

(30) $$\left\{\begin{array}{c} \frac{1}{r}u^{\prime }\left(r\right)+u^{\prime \prime }\left(r\right)=-\frac{\lambda^{2}\left(1+\delta \right|u^{\prime }\left(r\right){|}^{2})}{{\left(1-u\left(r\right)\right)}^{2}}\\ u\left(R\right)=0,\;\;\;\;u^{\prime }\left(0\right)=0,\;\;\;0<u\left(r\right)<d. \end{array}\right.$$

Furthermore, putting (5) in (30), the (6) holds. Accordingly, (30) becomes

(31) $$\left\{\begin{array}{c} u^{\prime \prime }\left(r\right)+\frac{1}{r}u^{\prime }\left(r\right)=-{\theta |E|}^{2}\left(1+\delta \right|u^{\prime }\left(r\right){|}^{2})\\ u\left(R\right)=0,\;\;\;\;u^{\prime }\left(0\right)=0,\;\;\;\theta \in R^{+},\;\;\;\;0<u\left(r\right)<d. \end{array}\right.$$

As observed in [24,48], E on the membrane is locally orthogonal to the straight-line tangent to the membrane’s profile of. Thus, |E| can be considered locally proportional to the mean curvature K(r,u(r)) of the membrane (see (7)). Of course, the greater the |E|, the greater the deformation of the membrane. Therefore, we can assume that |E|∝K(r,u(r)); based on this premise, we can write

(32) |E|=μ(r,u(r),λ)K(r,u(r))

in which μ(r,u(r),λ) represents the function of proportionality denoted as [24,48]

(33) $$\mu \left(r,u\left(r\right),\lambda \right)=\frac{\lambda }{\left(1-u\left(r\right)-d^{*}\right)}$$

where $\mu \left(r,u\left(r\right)\right)\in C^{0}\left(A\right)$, and A=[−R,R]×[0,1).

**Remark** **5.** *Usually, the models closest to the physical reality of MEMS are highly complex and cannot be faced analytically. It is thus necessary to operate with simplifications in the geometry of the devices, thereby obtaining simplified models that can be studied analytically. In other words, the results obtained from studying* (8) *will hardly agree with the experimental data. However, they will give an excellent qualitative contribution to the device’s behavior even if it is characterized by a simplified geometry.*

**Remark** **6.** *From* (6)*, we can observe that $\lambda^{2}$ is directly proportional to ${\left|E\right|}^{2}$ and therefore to $V^{2}$. Then, $\lambda^{2}$ is a bounded parameter, because a minimum value of V is required to overcome the mechanical inertia of the membrane (see below). On the other hand, the value of V cannot increase indefinitely because the intended use of the device fixes its maximum admissible value. Then, $\lambda^{2}$ is also limited by the intended use of the device.*

*Analytically, θ has no limitations except that it must be non-zero. However, as we will see below, the $\theta \lambda^{2}$ product will be subject to specific limitations due to problems related to the convergence of the numerical procedures used for recovering the membrane profile in the presence/absence of ghost solutions.*

*As far as μ(r,u(r),λ) is concerned, no apparent limitations to the values that can be attributed have appeared.*

Moreover, from (32), considering both (7) and (33), we obtain

(34) $${\left|E\right|}^{2}=\frac{1}{4}\frac{\lambda^{2}}{{\left(1-u\left(r\right)-d^{*}\right)}^{2}}{\left(u^{\prime \prime }\left(r\right)+\frac{1}{r}u^{\prime }\left(r\right)\right)}^{2}$$

so that (31) becomes

(35) $$\left\{\begin{array}{c} u^{\prime \prime }\left(r\right)+\frac{1}{r}u^{\prime }\left(r\right)=-\frac{\theta \lambda^{2}}{4{\left(1-u\left(r\right)-d^{*}\right)}^{2}}{\left(u^{\prime \prime }\left(r\right)+\frac{1}{r}u^{\prime }\left(r\right)\right)}^{2}\left(1+\delta \right|u^{\prime }\left(r\right){|}^{2})\\ u\left(R\right)=0,\;\;\;\;u^{\prime }\left(0\right)=0,\;\;\;\theta \in R^{+},\;\;\;\;0<u\left(r\right)<d. \end{array}\right.$$

Additionally, from (35), (8) follows because $u^{\prime \prime }\left(r\right)+\frac{1}{r}u^{\prime }\left(r\right)\neq 0$ (see [23]). It is a special case of the following general problem:

(36) $$\left\{\begin{array}{c} u^{\prime \prime }\left(r\right)+F\left(r,u\left(r\right),u^{\prime }\left(r\right)\right)=0\\ u\left(b\right)=B,\;\;\;\;u^{\prime }\left(a\right)=m, \end{array}\right.$$

where $F\in C^{0}\left(\left(a,b\right]\times R\times R\right)$ and B,m∈R. In fact, if

(37) $$F\left(r,u\left(r\right),u^{\prime }\left(r\right)\right)=\frac{1}{r}u^{\prime }\left(r\right)+\frac{4{\left(1-u\left(r\right)-d^{*}\right)}^{2}}{\theta \lambda^{2}\left(1+\delta \right|u^{\prime }\left(r\right){|}^{2})},$$

(38) B=m=0

and

(39) b=R,a=0,

Equation (4) is easily achieved. The general formulation (36) is allowed to exploit two necessary lemmas (see Lemmas 1 and 2 in [24]) for achieving an algebraic inequality that, if satisfied, ensured the existence of the solution for (8). For the simplicity for reading, we report this critical result of the existence [48].

## 4. On the Existence of At Least One Solution

**Theorem** **1.** *Let us consider Problem* (8)*. In addition, let us take into account two functions continuously differentiable twice, $u_{1}\left(r\right)$ and $u_{2}\left(r\right)$, both defined on [0,R], in order that $u_{1}\left(r\right)<u_{2}\left(r\right)$. Furthermore, let us suppose that*

(40) $$u_{1}^{\prime \prime }\left(r\right)+\frac{1}{r}u_{1}^{\prime }\left(r\right)+\frac{4{\left(1-u_{1}\left(r\right)-d^{*}\right)}^{2}}{\theta \lambda^{2}\left(1+\delta \right|u_{1}^{\prime }\left(r\right){|}^{2})}>0$$

*and*

(41) $$u_{2}^{\prime \prime }\left(r\right)+\frac{1}{r}u_{2}^{\prime }\left(r\right)+\frac{4{\left(1-u_{2}\left(r\right)-d^{*}\right)}^{2}}{\theta \lambda^{2}\left(1+\delta \right|u_{2}^{\prime }\left(r\right){|}^{2})}>0$$

*for r∈(0,R). If $\frac{1}{r}u^{\prime }\left(r\right)+\frac{4{\left(1-u\left(r\right)-d^{*}\right)}^{2}}{\theta \lambda^{2}\left(1+\delta \right|u^{\prime }\left(r\right){|}^{2})}$ is a continuous function (except for r=0), which satisfies the Lipschitz condition in U×(−∞,+∞), with*

(42) $$U=\{\left(r,u\right)\;:\;0<r<R\;\;and\;\;u_{1}\left(r\right)\leq u\left(r\right)\leq u_{2}\left(r\right)\}$$

*and if*

(43) $$u_{1}^{\prime }\left(0\right)\geq u_{2}^{\prime }\left(0\right)$$

*and*

(44) $$u_{1}\left(R\right)=u_{2}\left(R\right)=0$$

*with*

(45) $$\theta \lambda^{2}>\frac{2d*2R^{2}}{k\epsilon_{0}V^{2}\left(1+\delta {\left(\frac{k\epsilon_{0}V^{2}r}{d*2R^{2}}\right)}^{2}\right)},$$

*thus, Problem* (8) *admits at least one solution.*

**Proof** **of** **Theorem 1.** See [48]. □

**Figure 2:**
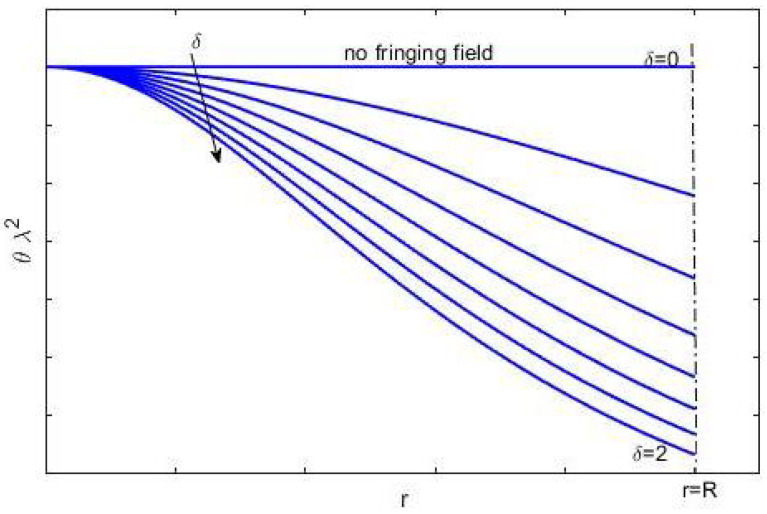
A graphical representation of (8) when δ changes; the forbidden area is located below each curve, while the permitted area is highlighted above each curve.

**Remark** **7.** (45) *represents the algebraic condition ensuring the existence of at least one solution for* (8). (45) *makes sense if the device is in operation (V≠0). If on the plane $\theta \lambda^{2}-r$, we represent* (45) *in the form of an equation, and we obtain a curve of the type shown in Figure 2. Above the curve is the area of the plane that satisfies* (45)*, while below the curve is the forbidden area. Further, if we take into account the limitation*

(46) 0≤δ<2

*obtained in [64], the trends of $\theta \lambda^{2}$, varying by δ, for 0≤r≤R are displayed in Figure 2, which indicates that as δ increases, the curve subsides, increasing the allowed area while decreasing the forbidden area. This effect becomes more evident from the axis of symmetry toward the device’s edges where the fringing effect is most evident.*

Then, following Remark 7, Figure 2 could be an indicative criterion for choosing the material for the membrane once the intended use of the device has been chosen. In other words, once *V* is fixed and quantified, the effect due to the fringing field (i.e., δ) and the allowed area on the $\theta \lambda^{2}-r$ plane is immediately visible, from which it is possible to select the most suitable range of values of $\theta \lambda^{2}$. Therefore, by (11), *T* is computable. Fixed δ and all materials that allow $\theta \lambda^{2}$ below the curve have to be excluded.

**Remark** **8.** *Note that the criterion above mentioned is purely theoretical as the differential analytical model proposed in this work, even if starts from physical considerations found in industrial reality (curvature of the membrane locally proportional |E|) has not yet had an experimental confirmation (not even in terms of hardware prototyping). Therefore, in the future, it would be appropriate to proceed with software simulations of materials more adherent to industrial reality such as graphene or the highly stressed silicon nitride in order to carry out a hardware prototyping of sure industry interest.*

**Remark** **9.** *From the work in [65], we can extrapolate the following important result:*

(47) $$H>\sqrt[{6}]{\frac{\theta \lambda^{2}}{2R^{2}\left(2-\delta \right)\left(1-d^{*}\right)}}$$

*which makes sense if only if δ<2. Obviously, for δ=1 no problem is encountered. However, for δ=2, it follows that H→+∞. However, to say that H→+∞ means to say that, given the symmetry of the membrane with respect to the origin of the Cartesian axes, at the boundary of the lower plate, the membrane adhere to the lateral surface of the device (unwanted harmful effect). Therefore, the value δ=2, avoided mathematically, must also be physically avoided.*

**Remark** **10.** *We note that the term due to the fringing field in the model is*

(48) $$\frac{\lambda^{2}\delta {\left|u^{\prime }\left(r\right)\right|}^{2}}{{\left(1-u\left(r\right)\right)}^{2}}=\xi {\left|E_{fringing\;field}\right|}^{2},\;\;\;\;\xi \in R$$

*where*

(49) $$\frac{\lambda^{2}}{{\left(1-u\left(r\right)\right)}^{2}}\propto {\left|E\right|}^{2}$$

*and the following dimensionless term,*

(50) $$\delta |u^{\prime }{\left(r\right)|}^{2}$$

*weighs the fringing field phenomenon. Then, the electrostatic force due solely to the fringing field holds:*

(51) $${\left[f_{el}\right]}_{fringing\;field}=\frac{0.5\epsilon_{0}\pi R^{2}\lambda^{2}\delta {\left|u^{\prime }\left(r\right)\right|}^{2}}{{\left(1-u\left(r\right)\right)}^{2}}$$

*considering that $|u^{\prime }{\left(r\right)|}^{2}<H^{2}=146^{2}$ [48], we can write:*

(52) $${\left[f_{el}\right]}_{fringing\;field}<\frac{296174\cdot 10^{-12}R^{2}\lambda^{2}\delta }{d^{2}}.$$

*Moreover, considering condition (11) and subbstituting the usual values for each physical parameter, we easily achieve*

(53) $${\left[f_{el}\right]}_{fringing\;field}<\frac{5242279\cdot 10^{-24}\delta }{d^{3}Td*2}$$

*which represents the link between the electrostatic force due to the fringing field and the δ parameter which weighs the effect due to the fringing field. Finally, we observe that the greater the mechanical tension of the membrane (term T in the denominator), the lower the effect due to the fringing field will be.*

## 5. A Well-Known Result of Uniqueness

**Theorem** **2.** *If all the hypotheses of Theorem 1 concerning* (8) *are satisfied, and $u_{1}\left(r\right)$ and $u_{2}\left(r\right)$ together satisfy the assigned boundary conditions, then the uniqueness of solution u(r), such that $u_{1}\left(r\right)\leq u\left(r\right)\leq u_{2}\left(r\right)$, is not guaranteed.*

**Proof** **of** **Theorem 2.** See [48] □

The uniqueness result proved in Theorem 2, although theoretically interesting, has a defect in that it does not depend on $\theta \lambda^{2}$ (i.e., the electromechanical properties of the material constituting the membrane). Therefore, in this paper, we present a new algebraic condition that ensures uniqueness for (8) involving $\theta \lambda^{2}$.

## 6. A New Condition Ensuring the Uniqueness of the Solution

The following result holds:

**Proposition** **1.** *The algebraic condition ensuring the uniqueness of the solution for* (8) *is the following:*

(54) $$\theta \lambda^{2}>4R\left(1+R\right)\left(1+\delta H^{2}\right)$$

*where $H=\sup_{r\in (0,R]}\left|u^{\prime }\left(r\right)\right|$.*

**Proof.**  As the proof to obtain (54) is quite extensive, please refer to its reading in the Appendix A. □

Note that $\sup_{r\in (0,R]}|u^{\prime }\left(r\right)|=|u^{\prime }\left(\pm R\right)|$. This result has been proved in [23] and confirmed numerically in [30]. On the other hand, due to reasons of symmetry, the points of Ω characterized by the greatest slope of the membrane profile can only be those located on ∂Ω.

**Remark** **11.** *Inequality* (54) *is very interesting because it expresses that the uniqueness of the solution for* (8) *is ensured if $\inf\{\theta \lambda^{2}\}$ depends on both the geometry of the device (i.e., radius R) and δ by that given algebraic combination. This makes physical sense because the greater the R, the greater the V that must be applied to overcome the inertia of the membrane (see* (11)*). Furthermore, the higher the δ, the higher the V must be overcome this effect and move the membrane.*

(45) and (54) represent the algebraic conditions that ensure, respectively, the existence and uniqueness of the solution for (8). With δ=0, in [64], it was proved that the condition of uniqueness weighed more than that of existence. We wonder if this result remains even in circular 2*D* geometry. The following section answers this question.

## 7. A New Algebraic Condition Ensuring Both the Existence and Uniqueness

The following results yield the following.

**Proposition** **2.** *Inequality* (45) *guarantees both the existence and uniqueness of the solution for* (8).

**Proof.**  As both (45) and (54) are verified, the following system makes sense:

(55) $$\left\{\begin{array}{c} \theta \lambda^{2}>\frac{2d*2R^{2}}{k\epsilon_{0}V^{2}\left(1+\delta {\left(\frac{k\epsilon_{0}V^{2}r}{d*2R^{2}}\right)}^{2}\right)}\;\;\;\;\left(\mathrm{existence}\right)\\ \theta \lambda^{2}>4R\left(1+R\right)\left(1+\delta H^{2}\right)\;\;\;\;\left(\mathrm{uniqueness}\right). \end{array}\right.$$

We observe that

(56) $$\frac{2d*2R^{2}}{k\epsilon_{0}V^{2}\left(1+\delta {\left(\frac{k\epsilon_{0}V^{2}r}{d*2R^{2}}\right)}^{2}\right)}>4R\left(1+R\right)\left(1+\delta H^{2}\right).$$

In fact, if absurdly

(57) $$\frac{2d*2R^{2}}{k\epsilon_{0}V^{2}\left(1+\delta {\left(\frac{k\epsilon_{0}V^{2}r}{d*2R^{2}}\right)}^{2}\right)}<4R\left(1+R\right)\left(1+\delta H^{2}\right),$$

we would easily obtain

(58) $$H>\sqrt{\frac{1}{\delta }\left(\frac{d^{*}R}{2\left(1+R\right)\epsilon_{0}V^{2}{\left(1+\delta \left(\frac{k\epsilon_{0}V^{2}r}{d*2R^{2}}\right)\right)}^{2}}-1\right)}.$$

Then, substituting in (58) the plausible values for each parameter, we will get $H>10^{12}$, which would contradict the fact that, in circular 2D geometry with fringing field, H=146 (see in [48]). Therefore, the existence condition also ensures the uniqueness of the solution for (8) so that each numerical solution that does not satisfy (45) represents a ghost solution. □

**Remark** **12.** *Unlike in 1D geometry with fringing field [64], in 2D circular geometry, the condition of existence also guarantees uniqueness. On the other hand, as proved in [48], even if uniqueness are not guaranteed, equilibrium configurations are obtained dangerously close to the upper disk of the device, which is fortunately stable.*

The model (8), as already specified previously, does not allow for obtaining explicit solutions. Therefore, to recover the membrane profiles, we resort to suitable numerical techniques for solving nonlinear BVPs with singularities. The following section presents a quick overview of the main characteristics of the numerical techniques used in this study.

## 8. Numerical Results

In this section, we present and discuss the numerical results obtained solving model (8) for recovering the membrane profile and the performances of the methods used to this end. More precisely, we have selected the following numerical approaches: the shooting method, Keller–Box scheme, and II/IV Lobatto IIIa formulas as a collocation procedure. A detailed discussion of these methods can be found in [32] with the only difference that in the present work, we used the secant method to obtain the zeros in the nonlinear equation generated by the shooting procedure. All simulations have been carried out by using the MATLAB *®* R2019a environment. To facilitate the discussion, the section is divided into five subsections: the first subsection discusses the criteria for avoiding the computation of ghost solutions. The second subsection addresses the convergence of the numerical procedures as a function of the parameter $\theta \lambda^{2}$. In the third subsection, remarks on the choice of the number of nodes are provided. A comparison of the recovered membrane profile retrieved by the selected numerical approach is provided in the four subsection. In the last subsection, we consider the values assumed by the applied voltage *V* to the device to overcome the inertia of the membrane, which is useful in practical applications.

### 8.1. Detection of Ghost Solutions

As mentioned previously, each numerical solution must satisfy (45); otherwise, it must be considered a ghost solution. Therefore, from (45), we can express

(59) $$\begin{array}{c} \theta \lambda^{2}>\frac{2d*2R^{2}}{k\epsilon_{0}V^{2}\left(1+\delta {\left(\frac{k\epsilon_{0}V^{2}r}{d*2R^{2}}\right)}^{2}\right)}=\frac{2d*6R^{6}}{k\epsilon_{0}V^{2}\left(d*4R^{4}+\delta k^{2}\epsilon_{0}^{2}V^{4}r^{2}\right)}, \end{array}$$

but, as r<R and $d*4R^{4}\ll 1$, (59) becomes

(60) $$\begin{array}{c} \theta \lambda^{2}>\frac{2d*2R^{2}}{k\epsilon_{0}V^{2}\left(1+\delta {\left(\frac{k\epsilon_{0}V^{2}r}{d*2R^{2}}\right)}^{2}\right)}=\frac{2d*6R^{6}}{k\epsilon_{0}V^{2}\left(d*4R^{4}+\delta k^{2}\epsilon_{0}^{2}V^{4}R^{2}\right)}. \end{array}$$

However, $d*4R^{4}\ll 1$, so (60) becomes

(61) $$\begin{array}{c} \theta \lambda^{2}>\frac{2d*6R^{6}}{k\epsilon_{0}V^{2}\left(1+\delta k^{2}\epsilon_{0}^{2}V^{4}R^{2}\right)} \end{array}$$

Again, combining (22), (19), and (10), and taking into account that u(r)<d, we easily obtain

(62) $$k=\frac{2u\left(r\right){\left(d-u\left(r\right)\right)}^{2}}{\left(1-{\left(\frac{r}{R}\right)}^{2}\right)\epsilon_{0}V^{2}}<\frac{2d{\left(d-u\left(r\right)\right)}^{2}}{\left(1-{\left(\frac{r}{R}\right)}^{2}\right)\epsilon_{0}V^{2}}$$

which substituted into (60), gives us

(63) $$\begin{array}{c} \theta \lambda^{2}\geq \frac{d*6R^{6}}{V^{2}\left(1+4\delta d^{2}{\left(d-u\left(r\right)\right)}^{4}\right)}. \end{array}$$

Therefore, any numerical procedure used, ∀δ∈[0,2) and under convergence conditions, produces a corresponding membrane profile. Then, we denote by $u_{j,\delta }\left(r\right)$ the profile of the membrane obtained by applying the j-th numerical procedure with a given value of δ. Then, for each numerical procedure *j* with a given value of δ, the value of $\theta \lambda^{2}$, starting from which convergence is ensured without ghost solutions, denoted by ${\left({\left(\theta \lambda^{2}\right)}_{conv-\;no\;ghost\;solutions}\right)}_{j,\delta }$, is the following:

(64) $$\begin{array}{c} {\left({\left(\theta \lambda^{2}\right)}_{conv-\;no\;ghost\;solutions}\right)}_{j,\delta }=\frac{d*6R^{6}}{V^{2}\left(1+4\delta d^{2}{\left(d-\max_{r}\left\{\max_{j}\left\{u_{j,\delta }\left(r\right)\right\}\right\}\right)}^{4}\right)}. \end{array}$$

Therefore,

(65) $$\left[{\left({\left(\theta \lambda^{2}\right)}_{conv-\;no\;ghost\;solutions}\right)}_{j,\delta },+\infty \right)$$

are the ranges of values ensuring convergence without ghost solutions, ∀j and ∀δ.

### 8.2. On the Convergence of the Numerical Procedures

As shown in previous works [30,31,32,60], $\theta \lambda^{2}$ that ensured the convergence of all the utilized numerical procedures (indicated by $\min{\left(\theta \lambda^{2}\right)}_{\mathrm{conv}}$) was obtained without considering the effects of the fringing field. Noticeably, if $\theta \lambda^{2}<\min{\left(\theta \lambda^{2}\right)}_{\mathrm{conv}}$, all the numerical procedures did not converge. Conversely, if $\theta \lambda^{2}>\min{\left(\theta \lambda^{2}\right)}_{\mathrm{conv}}$, all numerical procedures converged, and, in some cases, ghost solutions took place. Unlike the 1*D* case [65], wherein a decrease in the value of $\theta \lambda^{2}$ was highlighted, ensuring convergence as δ increases, whatever the numerical procedure used, the circular geometry 2*D* studied here does not show the same behavior. Rather, there is an increasing trend of these minimum values but, in any case, of extremely reduced amplitudes. Looking at the present work in more detail, starting from (8), we achieved ${\left(\theta \lambda^{2}\right)}_{\mathrm{conv}}$ exploiting, as in [65], the shooting technique by ode23 and ode45 (MatLab routines), Keller–Box scheme, and III/IV Stage Lobatto IIIa formulas by bpv4c and bpv5c (MatLab routines) when δ∈[0,2) increased, according to (46). Particularly, Table 1 and Table 2 highlight, as δ increases, the minimum value of $\theta \lambda^{2}$ ensuring convergence for each numerical procedure such that $\min{\left(\theta \lambda^{2}\right)}_{\mathrm{conv}}=10^{-3}$, which corresponds to the Keller–Box scheme with δ≥1. Then, ∀δ∈[0,2) the ranges of values relative to $\theta \lambda^{2}$ that do not allow convergence of the numerical procedures are shown in Table 3 and Table 4, which indicates that increasing the effect due to the fringing field increases the non-convergence intervals. Moreover, for each numerical procedure and ∀δ∈[0,2), the convergence intervals in the presence of ghost solutions (i.e., the numerical solutions that do not respect the condition (45)) have been obtained as shown in Table 5 and Table 6. Finally, Table 7 and Table 8 show the ranges of possible values for $\theta \lambda^{2}$, which ensure the convergence of all numerical procedures without ghost solutions (i.e., the numerical solutions obtained satisfy the condition (45)).

**Remark** **13.** *Notably, on increasing the effect due to the fringing field, we observe a shift toward lower values of the range of possible values for $\theta \lambda^{2}$ that ensures convergence (with/without ghost solutions). Furthermore, the numerical procedure that determines the minimum value of $\theta \lambda^{2}$ ensuring convergence for each numerical procedure without ghost solution, in our study, turned out to be the Keller–Box scheme (see Table 7).*

### 8.3. A Few Remarks on the Number of Nodes N

AAs far as the Keller–Box and Lobatto procedures (bpv4c and bpv5c, respectively) are concerned, a number of nodes, *N*, equal to 40 have been chosen not to deviate much from the number of nodes selected by ode23 (see Table 9). We observe that the Keller–Box scheme did not converge to the solution for values of *N* smaller. Moreover, the performance of Lobatto formula for values of *N* smaller did not reach the accuracy set by default, providing unreliable solutions (for example, deformed triangle-shaped membranes with a mesh consisting of an excessive number of points). Furthermore, note that the presented results were obtained by applying numerical methods of different convergence to show the efficiency and performance of the various numerical approaches in the existing literature. From a qualitative point of view, the recovered profiles are comparable even though tiny differences appear due to the different orders of accuracy of the proposed methods.

**Remark** **14.** (65) *is more general than the range of values shown in the previous tables because it is formulated ∀δ∈[0,2), while in the tables, the ranges shown refer to certain values of δ.*

### 8.4. The Recovering of the Membrane Profile: Performance of Numerical Procedures

**Figure 3:**
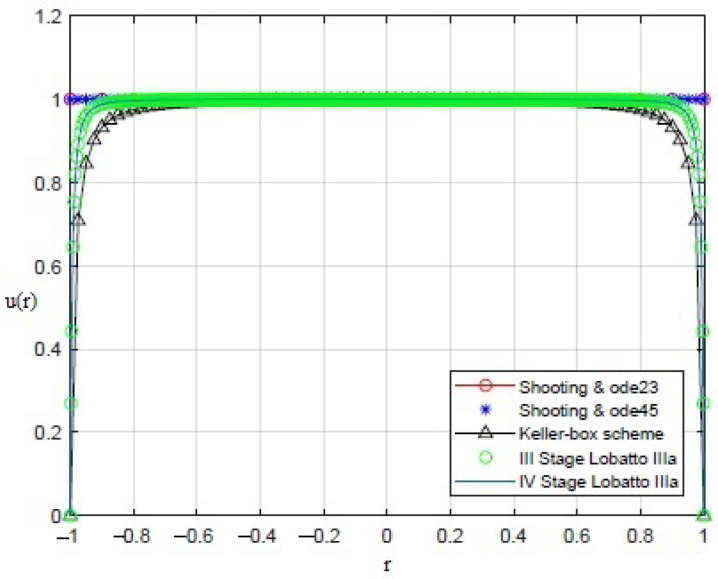
Recovering of u(r) for δ=0 and $\theta \lambda^{2}$ as reported in Table 1 and Table 2.

**Figure 4:**
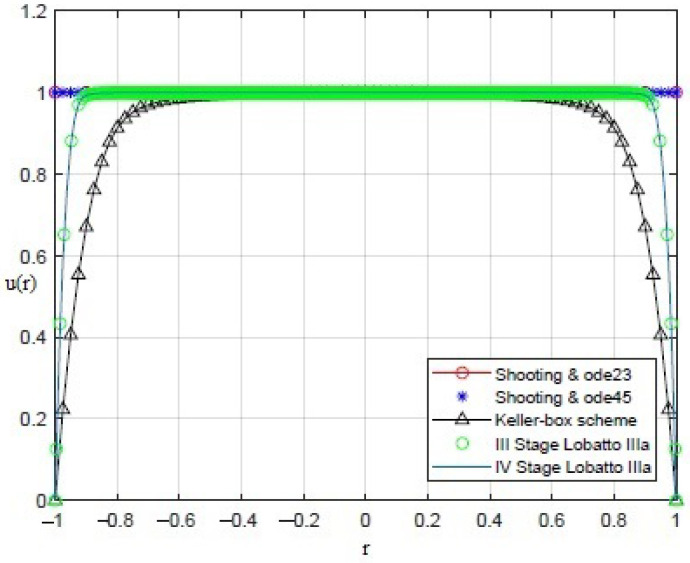
Recovering of u(r) for δ=0.5 and $\theta \lambda^{2}$ as reported in Table 1 and Table 2.

**Figure 5:**
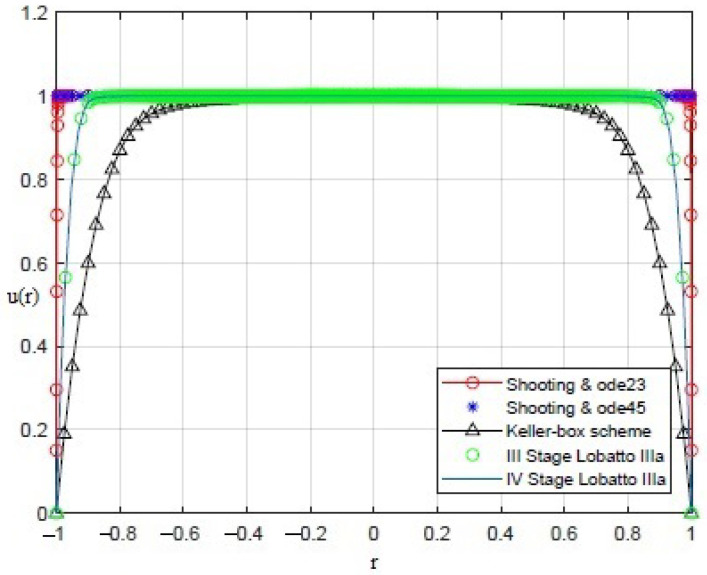
Recovering of u(r) for δ=1 and $\theta \lambda^{2}$ as reported in Table 1 and Table 2.

**Figure 6:**
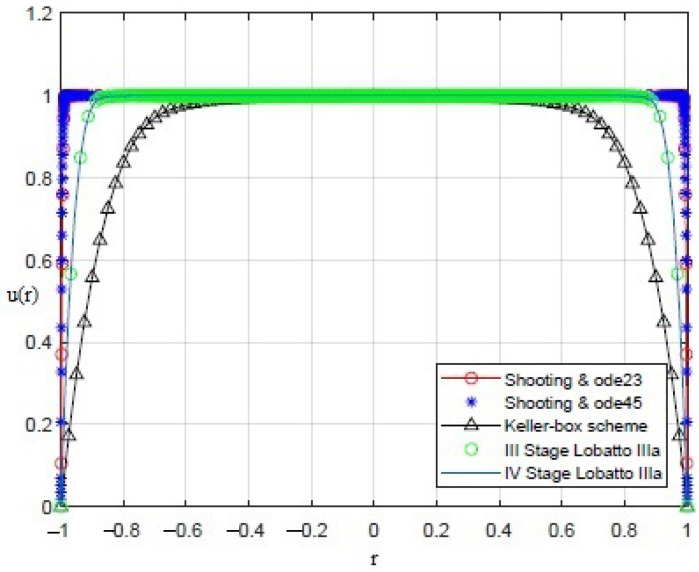
Recovering of u(r) for δ=1.5 and $\theta \lambda^{2}$ as reported in Table 1 and Table 2.

**Figure 7:**
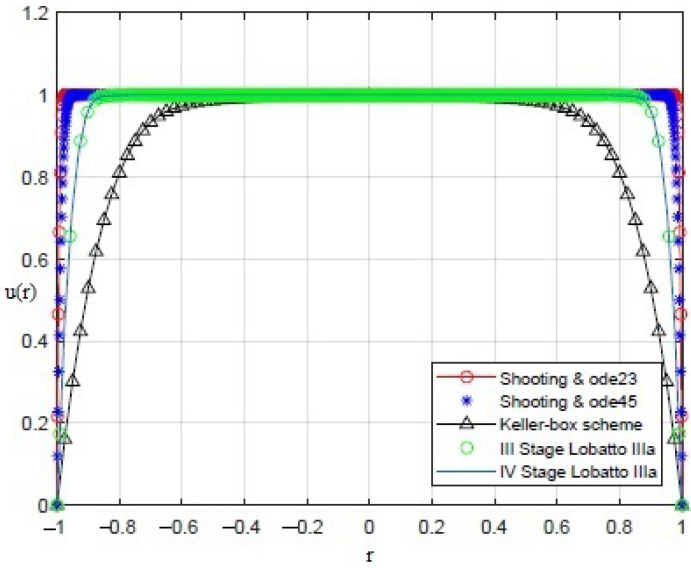
Recovering of u(r) for δ=1.99 and $\theta \lambda^{2}$ as reported in Table 1 and Table 2.

By applying the numerical procedures to (8) to recover the profile of the membrane, interesting results were obtained, as shown in Figure 3, Figure 4, Figure 5, Figure 6 and Figure 7. Particularly, Figure 3 depicts u(r) achieved by each numerical procedure when δ=0 with its corresponding value of $\theta \lambda^{2}$ as shown in Table 1 and Table 2. Similarly, in the other figures, for δ=0.5,1,1.5,1.99, we observe that, by ode23 with δ=0 and $\theta \lambda^{2}=1$, a displacement of the membrane is appreciated with a maximum value of about 0.4 at r=0, while for $\theta \lambda^{2}<1$, the solution remains almost constant (equal to 1) as if the whole membrane is moved up by an amount equal to 1. Finally, if $\theta \lambda^{2}\geq 10^{-15}$, the solution assumes values that are too high. Situations with completely similar results are obtained with ode45. However, note that a good recovering of the membrane is achieved with bpv4c when $\theta \lambda^{2}=10^{-5}$ and δ=0; with bpv4c when $\theta \lambda^{2}=10^{-6}$ and δ=1; and with bpv5c when $\theta \lambda^{2}=10^{-7}$ and δ=0.

**Remark** **15.** *As we will see below, the simulations performed by the numerical procedure did not show the recovering of the membrane profile in a regime of small displacements as proved in 1D geometry [65]. This allows us to highlight that the studied device could not be intended for biomedical applications (such as in drug delivery systems) wherein small displacements of the membrane are required. We also observe that the numerical methods used in this work are the most suitable for solving elliptic semi-linear differential problems with ordinary derivatives with singularities $\frac{1}{r}$. Among them, in terms of performance, the Keller–Box scheme method stands out, which pays a higher computational cost in the face of better performance. Therefore, Keller–Box method, even if it provides the best performance, could give problems in all those real-time applications where an extremely reduced membrane recovering time is required. Fortunately, such real-time applications are not numerous, so Keller–Box method is still attractive for many engineering applications.*

### 8.5. inf{V} to Overcome the Inertia of the Membrane with Fringing Field

As specified previously, if (45) is not satisfied, the numerical solution represents a ghost solution. Therefore, from (45), considering that r<R, we obtain

(66) $$\begin{array}{c} \frac{\theta \epsilon_{0}V^{2}}{d^{3}T}>\frac{d*2}{k\epsilon_{0}V^{2}\left(1+\delta {\left(\frac{k\epsilon_{0}V^{2}r}{d*2R^{2}}\right)}^{2}\right)}>\\ >\frac{2d*4R^{2}}{k\epsilon_{0}V^{2}\left(d*2+\delta k^{2}\epsilon_{0}^{2}V^{2}\right)}>\frac{2d*4R^{2}}{kV^{2}\left(d*2+\delta k^{2}V^{2}\right)} \end{array}$$

using which and exploiting (11), we achieve

(67) $$V>\underset{\inf\{V\}}{\underbrace{\sqrt[{4}]{\frac{d^{3}d*4T}{\theta \epsilon_{0}k\left(d*2+\delta k^{2}V^{2}\right)}}}}.$$

**Remark** **16.** (67)*, without fringing field (δ=0), is the same inequality obtained in [25] necessary to overcome the inertia of the membrane without fringing field. Conversely, when the fringing field phenomenon occurs, in circular 2D geometry and 1D geometry [65], the effect because of it appears in the denominator. In both kinds of geometry, the more intense the fringing field effect, the lower the V is to move the membrane because the lines of force of E near the edges of the device are noticeably curved outward, facilitating the deformation of the membrane. Thus, the movement of the membrane requires smaller values of V. This phenomenon is of great help in all those cases wherein the membrane MEMS device is a part of an electronic device for applications that, usually, are subject to reduced values of V. In these cases, the effects of the fringing field help move the membrane more easily, overcoming its inertia.*

### 8.6. Properties of the Material Constituting the Membrane & Intended Use of the Device in Non-Convergence Conditions

We preliminarily observe that ${\left|E\right|}^{2}\propto \frac{\lambda^{2}\left(1+\delta \right|u^{\prime }{\left(x\right)|}^{2}}{{\left(1-u\left(x\right)\right)}^{2}}$ such that

(68) $${\theta |E|}^{2}=\frac{\lambda^{2}\left(1+\delta \right|u^{\prime }\left(x\right){|}^{2})}{{\left(1-u\left(x\right)\right)}^{2}}.$$

Moreover, considering (11), (68) becomes

(69) $${\theta |E|}^{2}=\frac{4\epsilon_{0}^{2}V^{4}R^{4}\left(1+\delta \right|u^{\prime }\left(x\right){|}^{2})}{d^{6}T^{2}{\left(1-u\left(r\right)\right)}^{2}}$$

from which, multiplying both sides by $\lambda^{2}$ and considering again (11), we obtain

(70) $$\theta \lambda^{2}=\frac{4\epsilon_{0}^{2}V^{4}R^{4}\left(1+\delta \right|u^{\prime }\left(r\right){|}^{2})}{d^{6}T^{2}{\left(1-u\left(r\right)\right)}^{2}{\left|E\right|}^{2}}.$$

Furthermore, as

(71) $${\left|E\right|}^{2}{<\sup\{|E|}^{2}\}$$

it follows that

(72) $$\frac{1}{{\left|E\right|}^{2}}>\frac{1}{\sup\{|E{|}^{2}\}}$$

As

(73) 1−u(r)<1

it follows that

(74) $$\frac{1}{{\left(1-u\left(r\right)\right)}^{2}}>1.$$

Therefore, taking into account both (72) and (74), (70) becomes

(75) $$\theta \lambda^{2}>\frac{4\epsilon_{0}^{2}V^{4}R^{4}\left(1+\delta \right|u^{\prime }\left(r\right){|}^{2})}{d^{6}T^{2}{\sup\{|E|}^{2}\}}.$$

If the non-convergence conditions of each numerical procedure occur, it follows that

(76) $$\theta \lambda^{2}<\min_{j,\delta }\left\{{\left({\left(\theta \lambda^{2}\right)}_{conv}\right)}_{j,\delta }\right\},$$

where $\min_{j,\delta }\left\{{\left({\left(\theta \lambda^{2}\right)}_{conv}\right)}_{j,\delta }\right\}$ represents the minimum value of $\theta \lambda^{2}$ that ensures the convergence of all numerical procedures used for any value of δ∈[0,2). Therefore, (75) can be written as follows:

(77) $$\min_{j,\delta }\{{\left({\left(\theta \lambda^{2}\right)}_{conv}\right)}_{j,\delta }>\theta \lambda^{2}>\frac{4\epsilon_{0}^{2}V^{4}R^{4}\left(1+\delta \right|u^{\prime }\left(r\right){|}^{2})}{d^{6}T^{2}{\sup\{|E|}^{2}\}},$$

so that

(78) $$T>\sqrt{\frac{4\epsilon_{0}^{2}V^{4}R^{4}\left(1+\delta \right|u^{\prime }\left(r\right){|}^{2})}{d^{6}\min_{j,\delta }\{{\left({\left(\theta \lambda^{2}\right)}_{conv}\right)}_{j,\delta }{\sup\{|E|}^{2}\}}}.$$

Therefore, if we choose the intended use of the device, *V* is fixed so that, from (67), the effect due to the fringing field is not arbitrary. Furthermore, $\sup\{|E{|}^{2}\}$ inside the device also has a specific value. It follows that, in the conditions of non-convergence, all materials whose mechanical tension *T* satisfies the (78) must be avoided. Conversely, if the device has been chosen a priori (i.e., if the material constituting the membrane has been chosen a priori), then *T* is fixed, and, in the conditions of non-convergence, the intended uses of the device (i.e., the pairs ${\left\{V,\sup\{|E\right|}^{2}\}$) satisfying

(79) $$\frac{V^{4}}{\sup\{|E{|}^{2}\}}<\frac{\min_{j,\delta }\left\{{\left({\left(\theta \lambda^{2}\right)}_{conv}\right)}_{j,\delta }\right\}d^{6}T^{2}}{4\epsilon_{0}^{2}R^{4}\left(1+\delta \right|u^{\prime }\left(r\right){|}^{2})}$$

are not allowed.

**Remark** **17.** (78)*, without fringing field effects (δ=0), is the condition (73) achieved in [65]. This is because the membrane in circular geometry 2D, under symmetry conditions, can be considered as generated by the rotation of a 1D curveC, lying on the vertical plane xz, and rotating around the vertical axis z, making a complete rotation. Therefore, the electromechanical behavior of the membrane is the same on any vertical plane whose support is the rotation axis z. Similarly, *(79)*, without fringing field, becomes the condition (74) in [65]. However, we note that, in 2D circular geometry, unlike 1D geometry, the effects due to the fringing field is manifested not only by the presence of $\min_{j,\delta }\left\{{\left({\left(\theta \lambda^{2}\right)}_{conv}\right)}_{j,\delta }\right\}$ (as manifested in [65]), but also by the presence of $\delta |u^{\prime }{\left(r\right)|}^{2}$.*

### 8.7. Properties of the Material Constituting the Membrane & Intended Use of the Device in the Presence of Ghost Solutions

Indicating by $\min_{j,\delta }{\left\{{\left({\left(\theta \lambda^{2}\right)}_{conv}\right)}_{j,\delta }\right\}}_{limit}$ the value of $\theta \lambda^{2}$, in convergence conditions, below which ghost solutions occur, (69) with ghost solutions satisfies the following chain of inequalities:

(80) $$\begin{array}{c} \min_{j,\delta }\left\{{\left({\left(\theta \lambda^{2}\right)}_{conv}\right)}_{j,\delta }\right\}<\frac{4\epsilon_{0}R^{4}V^{4}\left(1+\delta \right|u^{\prime }\left(r\right){|}^{2})}{d^{6}T^{2}{\sup\{|E|}^{2}\}}<\min_{j,\delta }{\left\{{\left({\left(\theta \lambda^{2}\right)}_{conv}\right)}_{j,\delta }\right\}}_{limit} \end{array}$$

so that

(81) $$\begin{array}{c} \frac{d^{6}T^{2}\min_{j,\delta }\left\{{\left({\left(\theta \lambda^{2}\right)}_{conv}\right)}_{j,\delta }\right\}}{4\epsilon_{0}R^{4}\left(1+\delta \right|u^{\prime }\left(r\right){|}^{2})}<\frac{V^{4}}{\sup\{|E{|}^{2}\}}<\frac{d^{6}T^{2}\min_{j,\delta }{\left\{{\left({\left(\theta \lambda^{2}\right)}_{conv}\right)}_{j,\delta }\right\}}_{limit}}{4\epsilon_{0}R^{4}\left(1+\delta \right|u^{\prime }\left(r\right){|}^{2})}. \end{array}$$

Therefore, once the intended use of the device is chosen (i.e., once *V* satisfies (67) is fixed so that $\sup\{|E{|}^{2}\}$ is also fixed ), *T* that characterizes the material constituting the membrane must necessarily satisfy the chain of inequalities (81). Conversely, from (80), comes the following chain of inequalities:

(82) $$\begin{array}{c} \frac{d^{6}\min_{j,\delta }\left\{{\left({\left(\theta \lambda^{2}\right)}_{conv}\right)}_{j,\delta }\right\}{\sup\{|E|}^{2}\}}{4\epsilon_{0}RL^{4}V^{4}\left(1+\delta \right|u^{\prime }\left(r\right){|}^{2})}<\frac{1}{T^{2}}<\frac{d^{6}\min_{j,\delta }{\left\{{\left({\left(\theta \lambda^{2}\right)}_{conv}\right)}_{j,\delta }\right\}}_{limit}{\sup\{|E|}^{2}\}}{4\epsilon_{0}R^{4}V^{4}\left(1+\delta \right|u^{\prime }\left(r\right){|}^{2})}. \end{array}$$

Furthermore, in this case, once *T* has been set (i.e., once the membrane material has been selected), the operating parameters {V$,\sup\{|E{|}^{2}\}$} must be selected so that it is the (67) that the (82) conditions are both satisfied.

**Remark** **18.** *The range of $\theta \lambda^{2}$ that shows ghost solutions represents an electrostatic problem of great interest. In fact, the profiles recovered numerically are not able to satisfy the analytical model, thus requiring this range to be as small as possible. Therefore, from *(81)*, we achieve*

(83) $$\begin{array}{c} (\min_{j,\delta }\left\{{\left({\left(\theta \lambda^{2}\right)}_{conv}\right)}_{j,\delta }\right\}-\min_{j,\delta }{\left\{{\left({\left(\theta \lambda^{2}\right)}_{conv}\right)}_{j,\delta }\right\}}_{limit})<\\ <\frac{V^{4}}{\sup\{|E{|}^{2}\}}\frac{1}{T^{2}}\frac{4\epsilon_{0}R^{4}\left(1+\delta \right|u^{\prime }\left(r\right){|}^{2})}{d^{6}}. \end{array}$$

(83) *has a specific physical meaning. Once the device’s geometry is fixed (i.e., both R and d are fixed), the higher the T, the lower the $\theta \lambda^{2}$ that risk manifesting ghost will be solutions. In other words, the stiffer the membranes, the lower the risk of obtaining ghost solutions. Again, the higher the effects due to the fringing field, the greater the risk of obtaining ghost solutions. Finally, high values of the ratio $\frac{V^{4}}{\sup\{|E{|}^{2}\}}$ produces an increase in the area dedicated to ghost solutions by allocating the devices only to applications wherein reduced values of V are required to reduce the risk of ghost solutions.*

### 8.8. Properties of the Material Constituting the Membrane and Intended Use of the Device in Absence of Ghost Solutions

If *T* and ${\left\{V,\sup\{|E\right|}^{2}\}\}$ (such that *V* satisfies (67)) satisfy

(84) $$\frac{4\epsilon_{0}R^{4}V^{4}\left(1+\delta \right|u^{\prime }\left(r\right){|}^{2})}{d^{6}T^{2}{\sup\{|E|}^{2}\}}>\min_{j,\delta }{\left\{{\left({\left(\theta \lambda^{2}\right)}_{conv}\right)}_{j,\delta }\right\}}_{limit}$$

the device works in convergence condition without ghost solutions. Therefore, fixed ${\left\{V,\sup\{|E\right|}^{2}\}\}$, *T* must satisfy

(85) $$\frac{1}{T^{2}}>\frac{d^{6}\min_{j,\delta }{\left\{{\left({\left(\theta \lambda^{2}\right)}_{conv}\right)}_{j,\delta }\right\}}_{limit}{\sup\{|E|}^{2}\}}{4\epsilon_{0}R^{4}V^{4}\left(1+\delta \right|u^{\prime }\left(r\right){|}^{2})}.$$

Conversely, once *T* is fixed, ${\left\{V,\sup\{|E\right|}^{2}\}\}$ must be chosen in such a way that (85) is satisfied.

**Remark** **19.** (84) *represents an interesting limitation for the range of values of $\theta \lambda^{2}$. In fact, once the geometry of the device has been fixed, the membranes that are characterized by high mechanical stresses severely limit the presence of ghost solutions, allocating the device for all those applications with low electrical potential values.*

## 9. Conclusions

In this work, the membrane profile u(r) of an MEMS device with 2*D* circular geometry subject to an external electrical *V* applied between the disks has been numerically recovered. In the analytical model, we have considered that the electric field E, caused by the potential *V*, is locally orthogonal to the membrane’s tangent plane at the same point in a way that the principle according to which |E| results locally proportional to the membrane mean curvature, K(r,u(r)), turns out to be valid. In addition, for taking into account the effects due to the fringing field according to Pelesko and Driscoll’s theory, an addendum term has been joined in the considered analytical model. The term is weighted by a parameter depending on the square of the first derivative membrane profile amplitude. It takes into account the deformation of the lines of force of the field E near the edges of the device. Furthermore, an algebraic condition ensuring the uniqueness of the solution, which depends on the electromechanical properties of the membrane material, has also been demonstrated. Although the founded condition results to be less incisive than the condition of existence well known in the literature on these devices, it also ensures the solution’s uniqueness. The membrane profile has been recovered through several numerical procedures; we used the shooting method, the Keller–Box method, and the Lobatto IIIa stage III/IV formulas, comparing their performances. For this particular MEMS geometry, the links among (i) the electromechanical properties of the material constituting the membrane and (ii) the operational electrical parameters in conditions of convergence and non-convergence in the presence/absence of ghost solutions have been obtained. Furthermore, the ranges of the $\theta \lambda^{2}$ parameter, which ensures the convergence in the presence/absence of ghost solutions, have been obtained. Based on these results, a possible criterion for choosing the material constituting the membrane starting from *V* and vice versa in all device operating conditions has been provided. To conclude, we point out that the numerical results carried out in our study showed the clear superiority of the Keller–Box method compared with the other numerical techniques considered. However, because of its higher computational effort, the Keller–Box method could be less attractive for real-time applications.

Note that the numerical recovering in our work is a means to recognize any ghost solutions and understand the link between the properties of the material constituting the membrane and the intended use of the device in the different operating conditions. Therefore, a FEM analysis of the proposed analytical model is required (and developed in the near future) because it would allow us to obtain software formulations implementable in hardware for any prototyping of industrial use. However, in this work, we relied on the aforementioned numerical techniques because they currently represent the gold standard for this type of analytical models. Finally, it is worth nothing that concerning the material of the disks, in this work, we have considered metal plates. This arises from the fact that the analytical model here proposed derives from a well-known MEMS model with parallel metal plates proposed by D. Cassani et al. and cited in [23]. However, in the next future, it is our interest to propose analytical models closer to industrial reality, so the metal making up the disks should be replaced by more suitable materials (such as polysilicon).

## Figures and Tables

**Table 1:** Ranges of $\theta \lambda^{2}$ ensuring convergence (shooting and Keller–Box procedures).

δ	Shooting (ode 23)	Shooting (ode 45)	Keller–Box
0	${\left(\theta \lambda^{2}\right)}_{\mathrm{conv}}\in \left[10^{-14}\;+\infty \right)$	${\left(\theta \lambda^{2}\right)}_{\mathrm{conv}}\in \left[10^{-14}\;+\infty \right)$	${\left(\theta \lambda^{2}\right)}_{\mathrm{conv}}\in \left[10^{-3}\;+\infty \right)$
0.5	${\left(\theta \lambda^{2}\right)}_{\mathrm{conv}}\in \left[10^{-10}\;+\infty \right)$	${\left(\theta \lambda^{2}\right)}_{\mathrm{conv}}\in \left[10^{-10}\;+\infty \right)$	${\left(\theta \lambda^{2}\right)}_{\mathrm{conv}}\in \left[10^{-5}\;+\infty \right)$
1	${\left(\theta \lambda^{2}\right)}_{\mathrm{conv}}\in \left[10^{-10}\;+\infty \right)$	${\left(\theta \lambda^{2}\right)}_{\mathrm{conv}}\in \left[10^{-9}\;+\infty \right)$	${\left(\theta \lambda^{2}\right)}_{\mathrm{conv}}\in \left[10^{-3}\;+\infty \right)$
1.5	${\left(\theta \lambda^{2}\right)}_{\mathrm{conv}}\in \left[10^{-9}\;+\infty \right)$	${\left(\theta \lambda^{2}\right)}_{\mathrm{conv}}\in \left[10^{-9}\;+\infty \right)$	${\left(\theta \lambda^{2}\right)}_{\mathrm{conv}}\in \left[10^{-3}\;+\infty \right)$
1.99	${\left(\theta \lambda^{2}\right)}_{\mathrm{conv}}\in \left[10^{-8}\;+\infty \right)$	${\left(\theta \lambda^{2}\right)}_{\mathrm{conv}}\in \left[10^{-7}\;+\infty \right)$	${\left(\theta \lambda^{2}\right)}_{\mathrm{conv}}\in \left[10^{-3}\;+\infty \right)$

**Table 2:** Ranges of $\theta \lambda^{2}$ ensuring convergence (Three-/Four-Stage Lobatto IIIa).

δ	Three-Stage Lobatto IIIa (bpv4c)	Four-Stage Lobatto IIIa (bpv5c)
0	${\left(\theta \lambda^{2}\right)}_{\mathrm{conv}}\in \left[10^{-4}\;+\infty \right)$	${\left(\theta \lambda^{2}\right)}_{\mathrm{conv}}\in \left[10^{-6}\;+\infty \right)$
0.50	${\left(\theta \lambda^{2}\right)}_{\mathrm{conv}}\in \left[10^{-5}\;+\infty \right)$	${\left(\theta \lambda^{2}\right)}_{\mathrm{conv}}\in \left[10^{-5}\;+\infty \right)$
1	${\left(\theta \lambda^{2}\right)}_{\mathrm{conv}}\in \left[10^{-5}\;+\infty \right)$	${\left(\theta \lambda^{2}\right)}_{\mathrm{conv}}\in \left[10^{-5}\;+\infty \right)$
1.50	${\left(\theta \lambda^{2}\right)}_{\mathrm{conv}}\in \left[10^{-5}\;+\infty \right)$	${\left(\theta \lambda^{2}\right)}_{\mathrm{conv}}\in \left[10^{-5}\;+\infty \right)$
1.99	${\left(\theta \lambda^{2}\right)}_{\mathrm{conv}}\in \left[10^{-5}\;+\infty \right)$	${\left(\theta \lambda^{2}\right)}_{\mathrm{conv}}\in \left[10^{-5}\;+\infty \right)$

**Table 3:** Ranges of $\theta \lambda^{2}$ in conditions of non-convergence (shooting and Keller–Box procedures).

δ	Shooting (ode 23)	Shooting (ode 45)	Keller–Box
0	$\left[0,\;10^{-14}\right)$	$\left[0,\;10^{-14}\right)$	$\left[0,\;10^{-3}\right)$
0.5	$\left[0,\;10^{-10}\right)$	$\left[0,\;10^{-10}\right)$	$\left[0,\;10^{-5}\right)$
1	$\left[0,\;10^{-10}\right)$	$\left[0,\;10^{-9}\right)$	$\left[0,\;10^{-3}\right)$
1.5	$\left[0,\;10^{-9}\right)$	$\left[0,\;10^{-9}\right)$	$\left[0,\;10^{-3}\right)$
1.99	$\left[0,\;10^{-8}\right)$	$\left[0,\;10^{-7}\right)$	$\left[0,\;10^{-3}\right)$

**Table 4:** Ranges of $\theta \lambda^{2}$ in conditions of non-convergence (Three-/Four-Stage Lobatto IIIa).

δ	Three-Stage Lobatto IIIa (bpv4c)	Four-Stage Lobatto IIIa (bpv5c)
0	$\left[0,\;10^{-4}\right)$	$\left[0,\;10^{-6}\right)$
0.50	$\left[0,\;10^{-5}\right)$	$\left[0,\;10^{-5}\right)$
1	$\left[0,\;10^{-5}\right)$	$\left[0,\;10^{-5}\right)$
1.50	$\left[0,\;10^{-5}\right)$	$\left[0,\;10^{-5}\right)$
1.99	$\left[0,\;10^{-5}\right)$	$\left[0,\;10^{-5}\right)$

**Table 5:** Ranges of $\theta \lambda^{2}$ ensuring convergence with ghost solutions (shooting and Keller–Box procedures).

δ	Shooting (ode 23)	Shooting (ode 45)	Keller–Box
0	$\left[10^{-14},\;0.639\right)$	$\left[10^{-14},\;0.633\right)$	$\left[10^{-3},\;0.721\right)$
0.5	$\left[10^{-10},\;0.627\right)$	$\left[10^{-10},\;0.625\right)$	$\left[10^{-5},\;0.716\right)$
1	$\left[10^{-10},\;0.614\right)$	$\left[10^{-9},\;0.618\right)$	$\left[10^{-3},\;0.709\right)$
1.5	$\left[10^{-9},\;0.611\right)$	$\left[10^{-9},\;0.612\right)$	$\left[10^{-3},\;0.703\right)$
1.99	$\left[10^{-8},\;0.599\right)$	$\left[10^{-7},\;0.603\right)$	$\left[10^{-3},\;0.694\right)$

**Table 6:** Ranges of $\theta \lambda^{2}$ ensuring convergence with ghost solutions (Three-/Four-Stage Lobatto IIIa).

δ	Three-Stage Lobatto IIIa (bpv4c)	Four-Stage Lobatto IIIa (bpv5c)
0	$\left[10^{-4},\;0.693\right)$	$\left[10^{-6},\;0.698\right)$
0.50	$\left[10^{-5},\;0.686\right)$	$\left[10^{-5},\;0.691\right)$
1	$\left[10^{-5},\;0.679\right)$	$\left[10^{-5},\;0.684\right)$
1.50	$\left[10^{-5},\;0.672\right)$	$\left[10^{-5},\;0.677\right)$
1.99	$\left[10^{-5},\;0.668\right)$	$\left[10^{-5},\;0.669\right)$

**Table 7:** Ranges of $\theta \lambda^{2}$ ensuring convergence without ghost solutions (shooting and Keller–Box procedures).

δ	Shooting (ode 23)	Shooting (ode 45)	Keller–Box
0	[0.639,+∞)	[0.633,+∞)	[0.721,+∞)
0.5	[0.627,+∞)	[0.625,+∞)	[0.716,+∞)
1	[0.614,+∞)	[0.618,+∞)	[0.709,+∞)
1.5	[0.611,+∞)	[0.612,+∞)	[0.703,+∞)
1.99	[0.599,+∞)	[0.603,+∞)	[0.694,+∞)

**Table 8:** Ranges of $\theta \lambda^{2}$ ensuring convergence without ghost solutions (Three-/Four-Stage Lobatto IIIa).

δ	Three-Stage Lobatto IIIa (bpv4c)	Four-Stage Lobatto IIIa (bpv5c)
0	[0.693,+∞)	[0.698,+∞)
0.50	[0.686,+∞)	[0.691,+∞)
1	[0.679,+∞)	[0.684,+∞)
1.50	[0.672,+∞)	[0.677,+∞)
1.99	[0.668,+∞)	[0.669,+∞)

**Table 9:** Number of bondes for each numerical technique.

δ	Shooting (ode 23)	Shooting (ode 45)	Keller-Box	Three-Stage Lobatto IIIa (bpv4c)	Four-Stage Lobatto IIIa (bpv5c)
0	11	40	40	40	40
0.5	11	40	40	40	40
1	64	40	40	40	40
1.5	63	125	40	40	40
1.99	58	101	40	40	40

## Data Availability

Our study does not report any data.

## Abbreviations

The following abbreviations are used in this manuscript:

Ω	bounded circular smooth domain
*d*	distance between the two parallel disks
*r*	radial coordinate
*R*	radius of the device
u(r)	profile of the membrane
*V*	external electrical voltage
*T*	radial mechanical tension of the membrane at rest
$\lambda^{2}$	parameter depending on *V* and *T*
δ	parameter that weighs the fringing field effect
E	electrostatic field
|E|	amplitude of the electrostatic field
K(r,u(r))	mean curvature
$d^{*}$	critical security distance
θ	factor of proportionality
$\epsilon_{0}$	permittivity of the free space
$f_{el}$	electrostatic force
$p_{el}$	electrostatic pressure
*p*	mechanical pressure
$u_{0}$	displacement in the center of the membrane
$C_{el}$	electrostatic capacitance
ρ	density
*h*	thickness
*Y*	Young modulus
ν	Poisson ratio
*D*	stiffness coefficient
μ(r,u(r),λ)	function of proportionality
$u_{1}\left(r\right)$, $u_{2}\left(r\right)$	auxiliary functions
*k*	constant of proportionality between *p* and $p_{el}$
*H*	$\sup_{r\in (0,1]}\left|u^{\prime }\left(r\right)\right|$
FEM	Finite Element Method
tol	tolerance for Brent procedure
TOL	tolerance for Keller–Box Scheme
RK	Runge–Kutta Methods
G(r,s)	Green function

## Appendix A. Proof of Proposition 1

We preliminarly observe that model (8), exploiting a suitable Green’s function G(r,s), can be rewritten in its equivalent integral formulation [66,67,68]:

(A1) $$u\left(r\right)=\int_{-R}^{R}G\left(r,s\right)F\left(r,u\left(r\right),u^{\prime }\left(r\right)\right)ds$$

and considering (37), (A1) becomes:

(A2) $$u\left(r\right)=\int_{-R}^{R}G\left(r,s\right)\left[\frac{u^{\prime }\left(r\right)}{r}+\frac{4{\left(1-u\left(r\right)-d^{*}\right)}^{2}}{\theta \lambda^{2}\left(1+\delta \right|u^{\prime }\left(r\right){|}^{2})}\right]ds.$$

As known, G(r,s), in our problem, assumes the following form [66]:

(A3) $$G\left(r,s\right)=\frac{\left(s+R)(R-r\right)}{2R}$$

if −R≤s≤r and

(A4) $$G\left(r,s\right)=\frac{\left(R-s)(r+R\right)}{2R}$$

when r<s≤R. In addition:

(A5) $$G_{r}\left(r,s\right)=-\frac{\left(s+R\right)}{2R}$$

if −R≤s<r and

(A6) $$G_{r}\left(r,s\right)=\frac{\left(R-s\right)}{2R}$$

when r<s≤R. Furthermore, it is easy to prove that [66]:

(A7) $$0\leq G\left(r,s\right)\leq \frac{R}{2}\;\;\;\forall r,s\in -R,R]$$

and

(A8) $$G_{r}\left(r,s\right)\leq \frac{1}{2}.$$

By contradiction, we assume that $u_{1}\left(r\right)$ and $u_{2}\left(r\right)$ are tho different solutions for (8), in order that $u_{1}\left(r\right)=T\left(u_{1}\left(r\right)\right)$ and $u_{2}\left(r\right)=T\left(u_{2}\left(r\right)\right)$. Thus,

(A9) $$u_{1}\left(r\right)=T\left(u_{1}\left(r\right)\right)=\int_{-R}^{R}G\left(r,s\right)\left[\frac{u_{1}^{\prime }\left(r\right)}{r}+\frac{4{\left(1-u_{1}\left(r\right)-d^{*}\right)}^{2}}{\theta \lambda^{2}\left(1+\delta \right|u_{1}^{\prime }\left(r\right){|}^{2})}\right]ds$$

and

(A10) $$u_{2}\left(r\right)=T\left(u_{2}\left(r\right)\right)=\int_{-R}^{R}G\left(r,s\right)\left[\frac{u_{2}^{\prime }\left(r\right)}{r}+\frac{4{\left(1-u_{2}\left(r\right)-d^{*}\right)}^{2}}{\theta \lambda^{2}\left(1+\delta \right|u_{2}^{\prime }\left(r\right){|}^{2})}\right]ds,$$

from which

(A11) $$u_{1}^{\prime }\left(r\right)=T\left(u_{1}\left(r\right)\right)=\int_{-R}^{R}G_{r}\left(r,s\right)\left[\frac{u_{1}^{\prime }\left(r\right)}{r}+\frac{4{\left(1-u_{1}\left(r\right)-d^{*}\right)}^{2}}{\theta \lambda^{2}\left(1+\delta \right|u_{1}^{\prime }\left(r\right){|}^{2})}\right]ds$$

and

(A12) $$u_{2}^{\prime }\left(r\right)=T\left(u_{2}\left(r\right)\right)=\int_{-R}^{R}G_{r}\left(r,s\right)\left[\frac{u_{2}^{\prime }\left(r\right)}{r}+\frac{4{\left(1-u_{2}\left(r\right)-d^{*}\right)}^{2}}{\theta \lambda^{2}\left(1+\delta \right|u_{2}^{\prime }\left(r\right){|}^{2})}\right]ds.$$

Therefore, taking into account also both (A7) and (A8), we can write:

(A13) $$\begin{array}{c} \left|\right|u_{1}\left(r\right)-u_{2}\left(r\right){\left|\right|}_{C^{1}\left(\left[-R,R\right]\right)}=\\ =\underset{r\in [-R,R]}{\sup}|u_{1}\left(r\right)-u_{2}\left(r\right)|+\underset{r\in [-R,R]}{\sup}\left|u_{1}^{\prime }\left(r\right)-u_{2}^{\prime }\left(r\right)\right|=\\ =\underset{r\in [-R,R]}{\sup}|\int_{-R}^{R}G\left(r,s\right)\left[\frac{u_{1}^{\prime }\left(r\right)}{r}+\frac{4{\left(1-u_{1}\left(r\right)-d^{*}\right)}^{2}}{\theta \lambda^{2}\left(1+\delta \right|u_{1}^{\prime }\left(r\right){|}^{2})}\right]ds-\\ -\int_{-R}^{R}G\left(r,s\right)\left[\frac{u_{2}^{\prime }\left(r\right)}{r}+\frac{4{\left(1-u_{2}\left(r\right)-d^{*}\right)}^{2}}{\theta \lambda^{2}\left(1+\delta \right|u_{2}^{\prime }\left(r\right){|}^{2})}\right]ds|+\\ +\underset{r\in [-R,R]}{\sup}|\int_{-R}^{R}G_{r}\left(r,s\right)\left[\frac{u_{1}^{\prime }\left(r\right)}{r}+\frac{4{\left(1-u_{1}\left(r\right)-d^{*}\right)}^{2}}{\theta \lambda^{2}\left(1+\delta \right|u_{1}^{\prime }\left(r\right){|}^{2})}\right]ds-\\ -\int_{-R}^{R}G_{r}\left(r,s\right)\left[\frac{u_{2}^{\prime }\left(r\right)}{r}+\frac{4{\left(1-u_{2}\left(r\right)-d^{*}\right)}^{2}}{\theta \lambda^{2}\left(1+\delta \right|u_{2}^{\prime }\left(r\right){|}^{2})}\right]ds|=\\ =\underset{r\in [-R,R]}{\sup}|\int_{-R}^{R}G\left(r,s\right)\{\frac{u_{1}^{\prime }\left(r\right)}{r}+\frac{4{\left(1-u_{1}\left(r\right)-d^{*}\right)}^{2}}{\theta \lambda^{2}\left(1+\delta \right|u_{1}^{\prime }\left(r\right){|}^{2})}-\\ -\frac{u_{2}^{\prime }\left(r\right)}{r}+\frac{4{\left(1-u_{2}\left(r\right)-d^{*}\right)}^{2}}{\theta \lambda^{2}\left(1+\delta \right|u_{2}^{\prime }\left(r\right){|}^{2})}\}ds|+\\ +\underset{r\in [-R,R]}{\sup}|\int_{-R}^{R}G_{r}\left(r,s\right)\{\frac{u_{1}^{\prime }\left(r\right)}{r}+\frac{4{\left(1-u_{1}\left(r\right)-d^{*}\right)}^{2}}{\theta \lambda^{2}\left(1+\delta \right|u_{1}^{\prime }\left(r\right){|}^{2})}-\\ -\frac{u_{2}^{\prime }\left(r\right)}{r}+\frac{4{\left(1-u_{2}\left(r\right)-d^{*}\right)}^{2}}{\theta \lambda^{2}\left(1+\delta \right|u_{2}^{\prime }\left(r\right){|}^{2})}\}ds|\leq \\ \leq \underset{r\in [-R,R]}{\sup}\int_{-R}^{R}\left|G\left(r,s\right)\right||\frac{u_{1}^{\prime }\left(r\right)}{r}+\frac{4{\left(1-u_{1}\left(r\right)-d^{*}\right)}^{2}}{\theta \lambda^{2}\left(1+\delta \right|u_{1}^{\prime }\left(r\right){|}^{2})}-\\ -\frac{u_{2}^{\prime }\left(r\right)}{r}+\frac{4{\left(1-u_{2}\left(r\right)-d^{*}\right)}^{2}}{\theta \lambda^{2}\left(1+\delta \right|u_{2}^{\prime }\left(r\right){|}^{2})}|ds+\\ +\underset{r\in [-R,R]}{\sup}\int_{-R}^{R}\left|G_{r}\left(r,s\right)\right||\frac{u_{1}^{\prime }\left(r\right)}{r}+\frac{4{\left(1-u_{1}\left(r\right)-d^{*}\right)}^{2}}{\theta \lambda^{2}\left(1+\delta \right|u_{1}^{\prime }\left(r\right){|}^{2})}-\\ -\frac{u_{2}^{\prime }\left(r\right)}{r}+\frac{4{\left(1-u_{2}\left(r\right)-d^{*}\right)}^{2}}{\theta \lambda^{2}\left(1+\delta \right|u_{2}^{\prime }\left(r\right){|}^{2})}|ds\leq \\ \leq \frac{R}{2}\underset{r\in [-R,R]}{\sup}\int_{-R}^{R}|\frac{u_{1}^{\prime }\left(r\right)}{r}-\frac{u_{2}^{\prime }\left(r\right)}{r}+\\ +\frac{4}{\theta \lambda^{2}}\left[\frac{{\left(1-u_{1}\left(r\right)-d^{*}\right)}^{2}}{\left(1+\delta |u_{1}^{\prime }\left(r\right){|}^{2}\right)}-\frac{{\left(1-u_{2}\left(r\right)-d^{*}\right)}^{2}}{\left(1+\delta |u_{2}^{\prime }\left(r\right){|}^{2}\right)}\right]|ds+\\ +\frac{1}{2}\underset{r\in [-R,R]}{\sup}\int_{-R}^{R}|\frac{u_{1}^{\prime }\left(r\right)}{r}-\frac{u_{2}^{\prime }\left(r\right)}{r}+\\ +\frac{4}{\theta \lambda^{2}}\left[\frac{{\left(1-u_{1}\left(r\right)-d^{*}\right)}^{2}}{\left(1+\delta |u_{1}^{\prime }\left(r\right){|}^{2}\right)}-\frac{{\left(1-u_{2}\left(r\right)-d^{*}\right)}^{2}}{\left(1+\delta |u_{2}^{\prime }\left(r\right){|}^{2}\right)}\right]|ds=\\ =\left(\frac{R}{2}+\frac{1}{2}\right)\underset{r\in [-R,R]}{\sup}\int_{-R}^{R}|\frac{u_{1}^{\prime }\left(r\right)}{r}-\frac{u_{2}^{\prime }\left(r\right)}{r}+\\ +\frac{4}{\theta \lambda^{2}}\left[\frac{{\left(1-u_{1}\left(r\right)-d^{*}\right)}^{2}}{\left(1+\delta |u_{1}^{\prime }\left(r\right){|}^{2}\right)}-\frac{{\left(1-u_{2}\left(r\right)-d^{*}\right)}^{2}}{\left(1+\delta |u_{2}^{\prime }\left(r\right){|}^{2}\right)}\right]|ds. \end{array}$$

We note that

(A14) $$\begin{array}{c} \frac{1}{1+\delta |u^{\prime }{\left(r\right)|}^{2}}=\frac{1+\delta |u^{\prime }{\left(r\right)|}^{2}-\delta {\left|u^{\prime }\left(r\right)\right|}^{2}}{1+\delta |u^{\prime }{\left(r\right)|}^{2}}\leq \\ \leq 1+\delta |u^{\prime }{\left(r\right)|}^{2}-\delta |u^{\prime }{\left(r\right)|}^{2}\leq 1+\delta {\left|u^{\prime }\left(r\right)\right|}^{2}, \end{array}$$

therefore, (A13), taking into account (A14), becomes

(A15) $$\begin{array}{c} \left|\right|u_{1}\left(r\right)-u_{2}\left(r\right){\left|\right|}_{C^{1}\left(\left[-R,R\right]\right)}\leq \\ \leq \left(\frac{R}{2}+\frac{1}{2}\right)\underset{r\in [-R,R]}{\sup}\{\int_{-R}^{R}\frac{1}{r}\left|u_{1}^{\prime }\left(r\right)-u_{2}^{\prime }\left(r\right)\right|ds+\\ +\frac{4}{\theta \lambda^{2}}\int_{-R}^{R}{\left(1-u_{1}\left(r\right)-d^{*}\right)}^{2}{\left(1+\delta H^{2}\right)}^{2}ds+\\ \frac{4}{\theta \lambda^{2}}\int_{-R}^{R}{\left(1-u_{2}\left(r\right)-d^{*}\right)}^{2}\left(1+\delta H^{2}\right)ds\}ds\leq \\ \leq \left(\frac{R}{2}+\frac{1}{2}\right)\underset{r\in [-R,R]}{\sup}\{\int_{-R}^{R}\frac{1}{r}\left|u_{1}^{\prime }\left(r\right)-u_{2}^{\prime }\left(r\right)\right|ds+\\ +\frac{4}{\theta \lambda^{2}}{\left(1+\delta H^{2}\right)}^{2}\int_{-R}^{R}\left[{\left(1-u_{1}\left(r\right)\right)}^{2}+{\left(1-u_{2}\left(r\right)\right)}^{2}\right]ds\}=\\ =\left(\frac{R}{2}+\frac{1}{2}\right)\underset{r\in [-R,R]}{\sup}\{\frac{2R}{r}\left|u_{1}^{\prime }\left(r\right)-u_{2}^{\prime }\left(r\right)\right|+\\ +\frac{8R}{\theta \lambda^{2}}\left(1+\delta H^{2}\right)\left[{\left(1-u_{1}\left(r\right)\right)}^{2}+{\left(1-u_{2}\left(r\right)\right)}^{2}\right]ds\}=\\ =\left(\frac{R}{2}+\frac{1}{2}\right)\frac{2R}{r}\underset{r\in [-R,R]}{\sup}\left|u_{1}^{\prime }\left(r\right)-u_{2}^{\prime }\left(r\right)\right|+\\ +\left(\frac{R}{2}+\frac{1}{2}\right)\frac{8R}{\theta \lambda^{2}}\left(1+\delta H^{2}\right)\underset{r\in [-R,R]}{\sup}\left[{\left(1-u_{1}\left(r\right)\right)}^{2}+{\left(1-u_{2}\left(r\right)\right)}^{2}\right]\leq \\ \leq 2\left(R+1\right)R\underset{r\in [-R,R]}{\sup}\left|u_{2}^{\prime }\left(r\right)-u_{1}^{\prime }\left(r\right)\right|+\\ +\frac{4R\left(R+1\right)\left(1+\delta H^{2}\right)}{\theta \lambda^{2}}\underset{r\in [-R,R]}{\sup}\left|u_{2}\left(r\right)-u_{1}\left(r\right)\right|. \end{array}$$

From (A15), to achieve a contradiction, it is necessary to write

(A16) $$\left\{\begin{array}{c} 2(R+1)R<1\\ \frac{4R\left(R+1\right)\left(1+\delta H^{2}\right)}{\theta \lambda^{2}}<1. \end{array}\right.$$

From (A16) we observe that

(A17) $$\frac{4R\left(R+1\right)\left(1+\delta H^{2}\right)}{\theta \lambda^{2}}>2\left(R+1\right)R.$$

In fact, if absurdly

(A18) $$\frac{4R\left(R+1\right)\left(1+\delta H^{2}\right)}{\theta \lambda^{2}}<2\left(R+1\right)R$$

we easily would obtain

(A19) $$H<\sqrt{\left(\frac{\theta \lambda^{2}}{2}-1\right)\frac{1}{\delta }}$$

which results an absurd condition because in absence of fringing field (i.e., δ=0), it follow that H→+∞. In other words, without fringing field the membrane profiles numerically recovered are symmetric with respect the axis r=0 (see [32]) putting in evidence that $\max\{|u^{\prime }\left(r\right)|\}$ corresponds to r=±R. Moreover [48],

(A20) $$\max\{|u^{\prime }\left(r\right)|\}<\sup\{|u^{\prime }\left(r\right)\left|\right\}=H=146.$$

Therefore, on x=±R, there is no risk of dangerous adhesion between the membrane and the vertical walls of the device that could generate unwanted electrostatic effects. This phenomenon could occur if $|u^{\prime }\left(\pm R\right)|\to +\infty$. From which, it follows that the algebraic condition of uniqueness for the solution for (8) is

(A21) $$\theta \lambda^{2}>4\left(R+1\right)R\left(1+\delta H^{2}\right)$$

(i.e., the algebraic inequality (54)).
